# Impact of polygenic risk communication: an observational mobile application-based coronary artery disease study

**DOI:** 10.1038/s41746-022-00578-w

**Published:** 2022-03-11

**Authors:** Evan D. Muse, Shang-Fu Chen, Shuchen Liu, Brianna Fernandez, Brian Schrader, Bhuvan Molparia, André Nicolás León, Raymond Lee, Neha Pubbi, Nolan Mejia, Christina Ren, Ahmed El-kalliny, Ernesto Prado Montes de Oca, Hector Aguilar, Arjun Ghoshal, Raquel Dias, Doug Evans, Kai-Yu Chen, Yunyue Zhang, Nathan E. Wineinger, Emily G. Spencer, Eric J. Topol, Ali Torkamani

**Affiliations:** 1 Scripps Research Translational Institute, La Jolla, CA 92037 USA; 2 Scripps Clinic, La Jolla, CA 92037 USA; 3 Department of Integrative Structural and Computational Biology, Scripps Research, La Jolla, CA 92037 USA; 4 Stanford University, Palo Alto, CA 94305 USA; 5 DNAVisit Corporation, San Diego, CA 92130 USA; 6 Personalized Medicine National Laboratory, Centro de Investigación y Asistencia en Tecnología y Diseño del Estado de Jalisco A.C, Guadalajara, Jalisco 44270 México

**Keywords:** Risk factors, Acute coronary syndromes, Predictive markers

## Abstract

We developed a smartphone application, *MyGeneRank*, to conduct a prospective observational cohort study (NCT03277365) involving the automated generation, communication, and electronic capture of response to a polygenic risk score (PRS) for coronary artery disease (CAD). Adults with a smartphone and an existing 23andMe genetic profiling self-referred to the study. We evaluated self-reported actions taken in response to personal CAD PRS information, with special interest in the initiation of lipid-lowering therapy. 19% (721/3,800) of participants provided complete responses for baseline and follow-up use of lipid-lowering therapy. 20% (*n* = 19/95) of high CAD PRS vs 7.9% (*n* = 8/101) of low CAD PRS participants initiated lipid-lowering therapy at follow-up (*p*-value = 0.002). Both the initiation of statin and non-statin lipid-lowering therapy was associated with degree of CAD PRS: 15.2% (*n* = 14/92) vs 6.0% (*n* = 6/100) for statins (*p*-value = 0.018) and 6.8% (*n* = 8/118) vs 1.6% (*n* = 2/123) for non-statins (*p*-value = 0.022) in high vs low CAD PRS, respectively. High CAD PRS was also associated with earlier initiation of lipid lowering therapy (average age of 52 vs 65 years in high vs low CAD PRS respectively, *p*-value = 0.007). Overall, degree of CAD PRS was associated with use of any lipid-lowering therapy at follow-up: 42.4% (*n* = 56/132) vs 28.5% (*n* = 37/130) (*p*-value = 0.009). We find that digital communication of personal CAD PRS information is associated with increased and earlier lipid-lowering initiation in individuals of high CAD PRS. Loss to follow-up is the primary limitation of this study. Alternative communication routes, and long-term studies with EHR-based outcomes are needed to understand the generalizability and durability of this finding.

## Introduction

The utility and effectiveness of polygenic risk scores (PRS) in health-decision making has been the recent subject of intense interest and debate^1–4^. Early studies exploring the behavioral impact of genetic risk found no significant influence on behavior, though they have generally been performed in a context where specific actionability was limited both due to the broad nature of direct-to-consumer genetic risk panels and the limited risk stratification conveyed by early polygenic risk estimates^5–7^. Contemporary, indication-specific and prospective studies on PRS utility are limited.

**Fig. 1:**
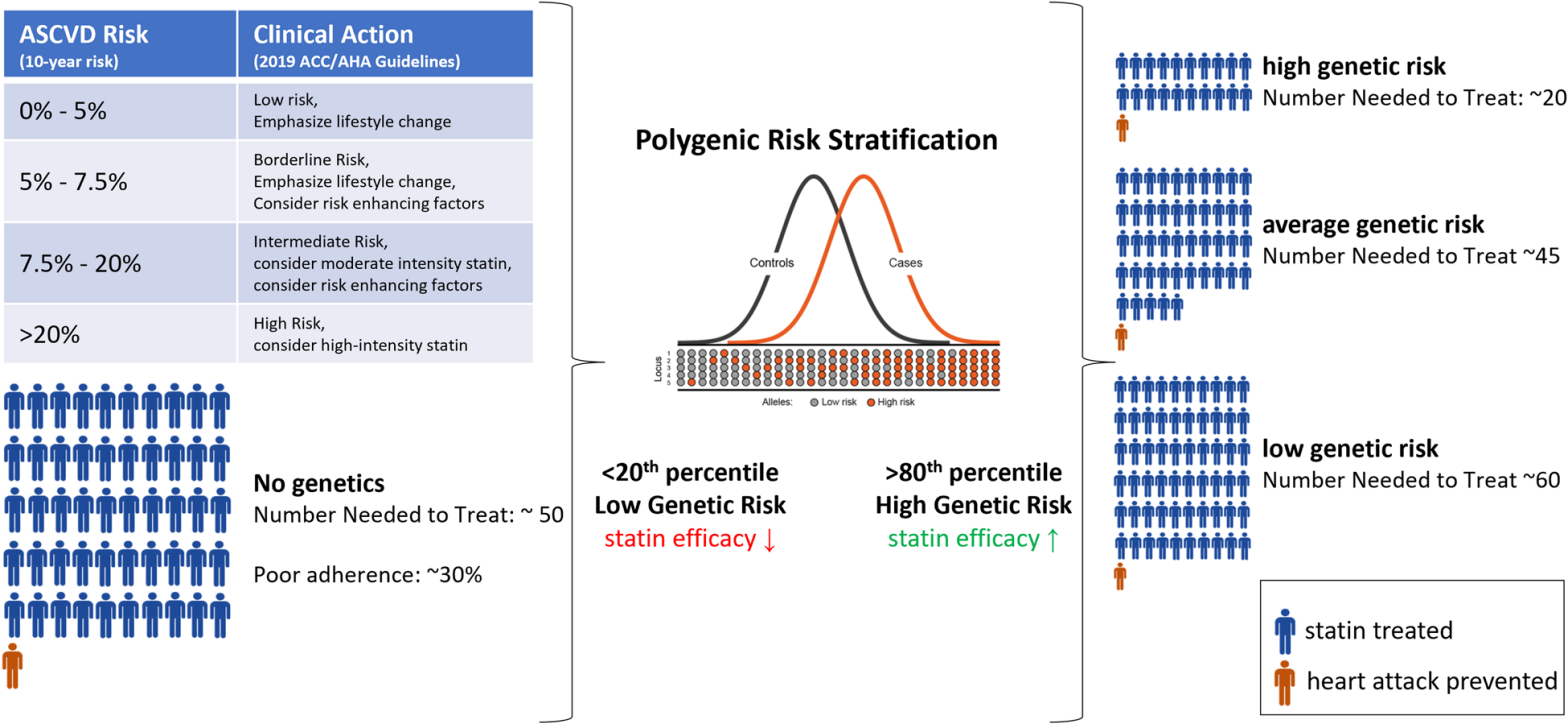
CAD PRS and statin efficacy. A CAD PRS can be introduced as a “risk enhancer” under current clinical guidelines (left), influencing preventive health decision-making through either re-classification of individuals across clinical risk tiers or, more often, by influencing the degree of therapeutic intervention used within each clinical risk tier. Each human figure represents a statin treated individual, with the figure in orange representing a heart attack prevented. The CAD PRS tiers and number needed to treat values depicted are derived from the landmark Mega et al. study^18^.

PRSs for coronary artery disease (CAD) are of special interest given that CAD is a highly heritable condition and the leading cause of preventable death in the developed world^8–11^. While there remains some debate regarding the precise utility of CAD PRSs in risk stratification^12–15^, high polygenic risk has been independently and repeatedly associated with enhanced benefit from lipid-lowering therapy^16–21^. This allows a CAD PRS to act as a “risk-enhancer” in any CAD preventive health-decision making scenario, beyond its contribution to risk stratification^11,22,23^ (Fig. 1). Furthermore, only ~30% of individuals who should use lipid-lowering therapies according to clinical guidelines do so, with no bias of use towards individuals with high polygenic risk, thus PRSs could be useful for simply encouraging adherence to clinical guidelines^24^.

Beyond multi-disease direct-to-consumer genetic studies^5–7^, a few focused clinical studies investigating patient actions in response to CAD PRS have been pursued^25,26^. These pilot studies were relatively small (*n* = 100–200) and investigated the clinical profiles of individuals receiving a clinical risk score vs a clinical risk score and PRS (rather than across PRS risk tiers). These studies were also recruited and conducted in clinical settings with direct clinical staff interaction in the communication of risk. Encouragingly, an improvement in clinical profiles was observed for all individuals receiving a PRS, regardless of their degree of genetic risk. A more recent Finnish study, Kardiokompassi, also enhanced with electronic risk visualization, has reported similar preliminary findings supporting a role in risk reduction broadly^27^.

Here we describe a prospective, app-based study, *MyGeneRank*, of CAD PRS communication, where participants join through self-referral from the community and provide informed consent electronically. Study participant response to CAD PRS information is self-initiated and self-guided with the support of interactive risk visualizers. Study participant response is gauged through electronic questionnaire. The objective of the study was to determine whether communication of a CAD PRS was associated with initiation of lipid-lowering therapy.

## Results

### Study participant and respondent baseline characteristics

Study participant and study respondent (those responding to follow-up) characteristics at baseline are presented in Table 1. For the subset of individuals providing complete information, we determined their precise clinical risk using the Pooled Cohort Equations^23^. The overall study population is largely comprised of clinically low-risk (<7.5% 10-year ASCVD risk based on PCE) individuals of European ancestry, with 73% of study participants and 63% of study respondents belonging to the clinical low-risk category at enrollment. Relative to the baseline study participant population, study respondents were more likely to be male (58% of study respondents vs 51% of study participants are male), of elevated age (47% vs 30% age >50 years old), non-smoker (2.6% vs 6.4% smokers), and currently on lipid-lowering medication (33% vs 23%). These differences enriched study respondents for individuals of intermediate clinical risk (20% of study respondents vs 13% of study participants) equally across CAD PRS tiers (Table 1). Study respondent characteristics are well-balanced across PRS tiers (Table 1). High CAD PRS respondents trend towards enrichment of male respondents and those already engaged in lipid-lowering therapy, but otherwise older study respondents with elevated PCE risk and high cholesterol levels trend towards enrichment in low CAD PRS respondents (Table 1).

**Table 1:** Study participant and respondent baseline characteristics.

	Study participants (*n* = 3484) % total (%low, %avg, %high)	Study respondents (*n* = 712) % total (%low, %avg, %high)	%Total participants vs %Total respondents (*p*-value)	%Low PRS respondent vs %High PRS respondent characteristics (*p*-value)
*Gender* (% female)	**49%** (51%, 49%, 46%)	**42%** (46%, 43%, 35%)	**<0.001**	0.04
*Age*				
18–29	17% (19%, 16%, 16%)	8.3% (5%, 9%, 9%)	<0.001	0.10
30–39	31% (31%, 32%, 31%)	24% (24%, 24%, 24%)	<0.001	0.46
40–49	22% (24%, 21%, 23%)	21% (21%, 21%, 24%)	0.41	0.27
50–59	**17%** (15%, 17%, 16%)	**22%** (21%, 24%, 18%)	**<0.001**	0.32
60–69	**9.6%** (8%, 10%, 11%)	**18%** (24%, 15%, 18%)	<**0.001**	0.11
70+	**3.2%** (3%, 4%, 3%)	**7.4%** (6%, 7%, 8%)	<**0.001**	0.28
*Ancestry*				
African American	1.9% (2.6%, 1.2%, 1.8%)	1.3% (3.7%, 0.2%, 2.3%)	0.14	0.25
American and Alaskan Native	1.6% (1.8%, 1.0%, 1.5%)	0.9% (2.2%, 0.7%, 0.7%)	0.08	0.17
Asian	5.5% (4.9%, 4.9%, 5.1%)	3.4% (2.2%, 3.7%, 3.1%)	0.01	0.33
Pacific Islander	0.6% (0.6%, 0.5%, 0.0%)	0.3% (0.0%, 0.0%, 0.8%)	0.16	0.15
White	93% (91%, 93%, 93%)	95% (97%, 92%, 95%)	0.01	0.24
Decline	1.5% (1.8%, 0.9%, 2.2%)	1.5% (0%, 1.5%, 2.3%)	0.50	0.04
*Hispanic or Latino Ethnicity*	6.8% (7.7%, 5.3%, 8.3%)	5.6% (8.0%, 4.7%, 6.9%)	0.72	0.34
*Body mass index*				
<20	6.2% (5.1%, 6.4%, 5.8%)	5.2% (5.1%, 4.8%, 6.9%)	0.18	0.27
20–25	39% (38%, 39%, 41%)	39% (37%, 40%, 39%)	0.39	0.34
25–30	33% (34%, 32%, 34%)	34% (39%, 32%, 35%)	0.35	0.23
30–35	13% (13%, 13%, 11%)	13% (13%, 14%, 10%)	0.39	0.21
≥35	9.4% (8.7%, 9.6%, 8.8%)	8.6% (5.8%, 9.2%, 9.2%)	0.25	0.15
*CAD Risk factors*				
high cholesterol	13% (12%, 11%, 15%)	15% (17%, 16%, 14%)	0.08	0.25
high blood pressure	4.6% (3.5%, 4.8%, 5.5%)	4.9% (5.1%, 4.5%, 5.3%)	0.36	0.46
smoking	**6.4%** (6.4%, 5.9%, 5.6%)	**2.6%** (2.2%, 2.5%, 3.1%)	**<0.001**	0.33
diabetes	2.7% (2.2%, 3.0%, 2.7%)	3.4% (2.2%, 3.8%, 2.3%)	0.15	0.47
active lifestyle	72% (73%, 72%, 70%)	77% (75%, 77%, 70%)	0.007	0.21
healthy diet	62% (61%, 63%, 63%)	63% (64%, 64%, 60%)	0.31	0.24
statin medication	**16%** (11%, 16%, 20%)	**27%** (23%, 27%, 30%)	**<0.001**	0.09
other lipid-lowering	**5.7%** (2.9%, 7.2%, 8.1%)	**9.8%** (5.4%, 12%, 11%)	<**0.001**	0.06
*10-year ASCVD Risk (PCE)*	(*n* = 990)	(*n* = 282)		
<5%	**73%** (74%, 72%, 74%)	**63%** (59%, 65%, 63%)	**0.001**	0.35
5–7.5%	10% (10%, 10%, 10%)	11% (16%, 10%, 11%)	0.62	0.19
7.5%–20%	**13%** (13%, 14%, 12%)	**20%** (20%, 20%, 18%)	**0.004**	0.40
>20%	3.7% (2.9%, 3.8%, 4.4%)	6% (4.9%, 4.8%, 8.9%)	0.16	0.19

Baseline characteristics of study participants and respondents in total and broken down by CAD PRS tier in parenthesis. Unadjusted *p*-values for proportion comparisons are presented in the two rightmost columns as determined using one-tailed two-proportion *z*-test. Significant differences are bolded. Relative to study participants, study respondents are enriched in older, non-smoking, individuals reporting use of lipid lowering therapy at baseline. No significant differences are observed in the baseline characteristics of study respondents across CAD PRS tiers. Responses are based on self-report.

### Study participant reactions

Of the 3800 study participants, 1053 (28%) provided their immediate reaction to learning their CAD PRS by expressing their degree of (dis)agreement with 12 statements. The statements where reactions differed significantly by CAD PRS category are presented in Table 2. All statements with large, standardized effect sizes are related to self-perceptions of risk: i.e., “My chances of developing Coronary Artery Disease are high” and “My genetics make it more likely that I will get coronary artery disease”—where low CAD PRS individuals expressed mild disagreement, average CAD PRS individuals were neutral, and high CAD PRS individuals expressed agreement with these statements. A small effect size was also observed for: “I worry a lot about developing Coronary Artery Disease”, where low and average CAD PRS individuals expressed mild disagreement, and high CAD PRS individuals were neutral in response to this statement. Reactions to other statements are provided in Supplementary Table 1. Overall, study participants agreed uniformly that they understood their risk, that they are able to reduce their risk, and that CAD is a serious condition.

**Table 2:** Study participant reactions.

Statement	*n*	Low CAD PRS	Average CAD PRS	High CAD PRS	Standardized effect size	Low vs high *P*-value
My chances of developing Coronary Artery Disease are high.	low = 69, avg = 165, high = 73	2.39 ± 0.14	3.13 ± 0.09	4.18 ± 0.13	0.63	8.8E−14
My genetics make it more likely that I will get Coronary Artery Disease.	low = 69, avg = 165, high = 73	2.48 ± 0.16	3.33 ± 0.09	4.23 ± 0.12	0.61	3.08E−13
My chances of getting Coronary Artery Disease are high.	low = 170, avg = 406, high = 152	2.25 ± 0.09	2.96 ± 0.06	3.86 ± 0.09	0.58	<1e−16
I am at risk of getting Coronary Artery Disease.	low = 170, avg = 406, high = 152	2.84 ± 0.1	3.53 ± 0.06	4.15 ± 0.08	0.49	1.11E−16
I worry a lot about developing Coronary Artery Disease.	low = 239, avg = 571, high = 224	2.44 ± 0.08	2.68 ± 0.05	3.09 ± 0.08	0.25	7.38E−08

Psychosocial responses showing significant difference across CAD PRS tiers. Average Likert scores plus standard errors of the means are displayed. Significance determined by Mann–Whitney *U* test. See Supplementary Table S3 for psychosocial responses showing no significant differences across CAD PRS tiers.

### Study participant intentions

Similarly, study participants were surveyed for their intended actions after receiving their CAD PRS. Specifically, participants were asked (1) “Now that you know your coronary artery disease genetic risk score, do you intend to make any changes to your use of statins?”, and (2) “Now that you know your coronary artery disease genetic risk score, do you intend to meet with a physician to discuss these results?” Study participants reported intentions to meet with their physician (2-fold difference in high vs low CAD PRS) and begin the use of statins (4-fold difference in high vs low CAD PRS) in strong association with their degree of genetic risk (Table 3).

**Table 3:** Study participant intentions.

Statement	Low CAD PRS (*n* = 240)	Average CAD PRS (*n* = 581)	High CAD PRS (*n* = 232)	Low vs high *P*-value
Now that you know your coronary artery disease genetic risk score, do you intend to make any changes to your use of statins? (% begin or increase use of statins)	5%	10.5%	19.0%	<0.00001
Now that you know your coronary artery disease genetic risk score, do you intend to meet with a physician to discuss these results? (% yes)	33.3%	40.8%	60.3%	<0.00001

Study participant intentions after receipt of their CAD PRS. Significance determined by one-tailed two proportion *z*-test of high vs low risk individuals.

### Study respondent actions

At 6-months follow-up or later, 253 study respondents proactively answered this survey within the study app while 459 study respondents answered the email survey. Follow-up times were 1-year on average for app-based respondents and 1.5 years on average for email-based respondents. We gauged the initiation and/or intensification of lipid-lowering in two ways: (1) by comparing responses relating to lipid-lowering therapy use at baseline and follow-up, and (2) by asking about attribution of changes in lipid-lowering to receipt of the PRS at follow-up.

For our primary analysis, we compared baseline and follow-up responses to two questions: (1) “Are you currently taking or have you previously taken a statin?” and (2) “Are you currently taking or have you previously taken any medication, other than a statin, used to treat high cholesterol?” The comparison of baseline vs follow-up responses is presented in Table 4. Study respondents of high CAD PRS and not taking a statin at baseline, initiated statin therapy at ~2.5-fold the rate of low CAD PRS study respondents (15.2% vs 6% statin initiation rate, *p*-value = 0.018). Similarly, study respondents of high CAD PRS and not taking non-statin lipid-lowering therapy at baseline, initiated non-statin lipid-lowering therapy at ~3-fold the rate of low CAD PRS individuals (6.8% vs 1.6% non-statin initiation rate, *p*-value 0.022). Discontinuation rates were low and not associated with degree of genetic risk (Table 4).

**Table 4:** Initiation and discontinuation of lipid-lowering therapy.

	Low CAD PRS (*n* = 132)		Average CAD PRS (*n* = 387)		High CAD PRS (*n* = 130)		Initiation rate (low vs high *p*-value)
	Follow-up		Follow-up		Follow-up		low, avg, high
	No	Yes	No	Yes	No	Yes	
*Statins*							
Baseline							
No	72.4%	***4.6%***	69%	***4.4%***	59%	***10.6%***	***6%***, ***6%***, ***15.2%*** (0.018)
Yes	0%	23%	2.1%	24.5%	0.8%	29.5%	
*Other Lipid-lowering*							
Baseline							
No	93.1%	***1.5%***	84.5%	***3.6%***	83.3%	***6.1%***	***1.6%***, ***4.1%***, ***6.8%*** (0.022)
Yes	3.8%	1.5%	5.9%	5.9%	3.8%	6.8%	

Confusion matrices depicting the % of study respondent reporting the use of statin and non-statin lipid-lowering at baseline and/or at follow-up. Values corresponding to lipid lowering initiators (“No” at baseline, “Yes” at follow-up) are bolded and italicized. Significance determined by one-tailed two proportion *z*-test of high vs low risk individuals. The rightmost initiation rate column provides percentages based only on those individuals reporting non-use of statins or other lipid-lowering therapy at baseline. The remaining columns report percentages based on the total population (total population including those individuals reporting use of lipid-lowering therapy at baseline). Significance determined by one-tailed two proportion *z*-test of high vs low risk individuals.

We also asked directly about lipid-lowering actions attributed to receipt of the CAD PRS through the following three questions: (1) “Did you meet with a physician to discuss your coronary artery disease risk score?,” (2) “After receiving your coronary artery disease genetic risk score, did you make any changes to your use of statins?,” and (3) “After receiving your coronary artery disease genetic risk score, did you make any changes to your use medications, other than statins, used to treat high cholesterol?” In response to this question, a clear difference in response to the PRS by level of engagement was apparent (Table 5). Proactive app-based responses displayed a significant relationship between attribution of changes in lipid-lowering therapy to receiving a CAD PRS, while email-based responses did not maintain this relationship. Study respondents reported that they met to discuss their results with their physician regardless of degree of risk, with a non-significant trend in association with degree of genetic risk. For high CAD PRS lipid lowering initiators, 58% (*n* = 11 of 19) report speaking with their physician about their results. For low CAD PRS lipid lowering initiators, 44% (*n* = 4 of 9) report speaking with their physician about their results.

**Table 5:** Attribution of lipid-lowering therapy changes.

Statement	Low CAD PRS (*n* = 66 app) (*n* = 100 email)	Average CAD PRS (*n* = 129 app) (*n* = 260 email)	High CAD PRS (*n* = 52 app) (*n* = 100 email)	Low vs high *P*-value (app) (email)
After receiving your coronary artery disease genetic risk score, did you make any changes to your use of statins? (% begin or increase use)	1.5% app-based 14% email-based	5.4% app-based 6.2% email-based	9.6% app-based 5% email-based	0.02 1.0
After receiving your coronary artery disease genetic risk score, did you make any changes to your use medications, other than statins, used to treat high cholesterol? (% begin or increase use)	0% app-based 5% email-based	3.8% app-based 4.3% email-based	11.8% app-based 6% email-based	0.002 0.38
Did you meet with a physician to discuss your coronary artery disease risk score? (% yes)	19.4% app-based 21% email-based	26.3% app-based 17.1% email-based	30.2% app-based 28.6% email-based	0.09 0.10

Study respondent attribution of actions at follow-up by mode of response. Significance determined by one-tailed two proportion *z*-test of high vs low risk individuals.

### Overall use of lipid-lowering therapies

Finally, we compare the rate of statin, non-statin, and combined lipid-lowering therapies at baseline and follow-up across study respondents (Table 6). While we observe a baseline statin usage rate of 27% overall, which is consistent with prior reports^24^, our results conflict with prior reports in that we observe a weak association of lipid-lowering with degree of genetic risk at baseline (Table 6). This association achieves statistical significance only for combined use of lipid-lowering therapies. At follow-up, a stronger and significant association of statin, non-statin, and combined lipid-lowering therapy use with degree of genetic risk is observed. Study respondents with high CAD PRS were ~1.4-fold more likely to report use of a statin at follow-up and ~4-fold more likely to report use of a non-statin lipid-lowering medication at follow-up, resulting in a ~1.5× difference in the rate of combined lipid-lowering therapy use in high vs low CAD PRS individuals (Table 6). When considering only study respondents not engaging in any lipid-lowering therapy at baseline, we observe a strong and significant association of lipid-lowering therapy initiation with degree of genetic risk: 7.9% (*n* = 101) vs 9.9% (*n* = 284) vs 20% (*n* = 95) of study respondents initiate any lipid-lowering therapy in low, average, and high CAD PRS individuals respectively (*p*-value = 0.002, two proportion z-test). The average age of study respondents who initiate lipid-lowering therapy also differs significantly (average age of 52 years old vs 65 years old in high CAD PRS vs low CAD PRS respectively, *p*-value = 0.007 by Mann–Whitney *U* Test). Overall lipid-lowering therapy initiation occurred at >2-fold the rate and >10 years earlier in study respondents receiving a high vs low CAD PRS.

**Table 6:** Changes in the use of lipid-lowering therapy.

	Low CAD PRS (*n* = 132)		Average CAD PRS (*n* = 387)		High CAD PRS (*n* = 130)		Low vs high *P*-value
	Baseline	Follow-up	Baseline	Follow-up	Baseline	Follow-up	(Baseline) (Follow-up)
Statins	23.1%	***27.7%***	26.6%	28.9%	30.3%	***40.2%***	0.09 **0.017**
Non-statin	5.4%	***3.1%***	11.9%	9.6%	10.6%	***12.9%***	0.06 **0.002**
Combined Lipid-lowering	23.1%	***28.5%***	29.2%	31.5%	33.3%	***42.4%***	0.03 **0.009**

Study respondent use of statin and non-statin lipid-lowering at baseline and follow-up. Values of most interest are bolded and italicized. An overall improved alignment of risk reducing interventions with degree of genetic risk is observed at follow-up. Significance determined by one-tailed two proportion *z*-test of high vs low risk individuals.

## Discussion

Here we report the results of a real-world, fully-digitized, and participant-centric approach to communication of CAD genetic risk via a PRS combined with clinical risk evaluation. We find that communication of a CAD PRS is associated with improved and earlier alignment of risk reducing interventions with degree of genetic risk. The rate of lipid-lowering therapy initiation (20%) in high CAD PRS individuals is remarkable given the overall low clinical risk profile of study participants, at least according to the Pooled Cohort Equations (PCE). We also find that CAD PRS information is perceived to be understandable, actionable, and does not induce health anxiety (see Table 2 and Supplementary Table 3). Overall, our study provides evidence that a CAD PRS could potentially be a useful tool for reinforcement of guidelines in a prevention setting.

Singular risk factors, like hypercholesterolemia or diabetes status can provide a basis for early lipid lowering initiation without achieving the typical PCE risk threshold of 7.5%. Similarly, it has been recently suggested that, an extreme CAD PRS could also provide a singular basis for early initiation of lipid lowering therapy^28^. As such, we evaluated whether such a basis existed in this study for the youngest high CAD PRS individuals (a 25-year-old male, 35-year-old-male, and 35-year-old-female) reporting initiation of lipid lowering therapy. The 25-year-old male scored an extreme 98th percentile CAD PRS and reported a non-HDL cholesterol of 189 mg/dL. In contrast, the 35-year-old male and female scored at the 82nd and 85th CAD PRS percentile, respectively, but both also report moderately high total cholesterol levels, a family history of heart attack for the male, and a diagnosis of diabetes for the female. Thus, of the three youngest lipid lowering initiators; one reports a definitive class I indication for the initiation of lipid lowering therapy (diabetes), one reports a likely class I indication for initiation of lipid lowering therapy (significant hyperlipidemia) along with an extreme CAD PRS, and the third reports a risk enhancer but does not meet guidelines for the initiation of lipid lowering therapy overall. Therefore, even in those lipid lowering initiators with the lowest PCE risk, communication of the CAD PRS did not induce major deviations from guideline recommendations. In most cases, communication of the CAD PRS served to reinforce guideline recommendations.

There are a series of important limitations which restrict the generalizability and significance of our findings. First, the study population consists of a convenience sampling of “early adopters,” predominantly of European ancestry, who have already generated their genetic profiles from 23andMe, and who may be more engaged in preventive health in comparison to the general population. For example, the enrichment of non-smoker study respondents supports the notion that the worried well and early adopters drive some of the statistical signal we observe. Further avenues for the delivery of CAD PRS information must be explored to generalize the impact to different communities.

Second, our study highlights some of the limitations of pure electronic communication of risk and provides some early indications that the association between lipid-lowering therapy and CAD PRS can be further amplified and generalized by deeper engagement with participants and/or with treating physicians as well as genetic counselors. For example, differences in attribution of response from app-based (proactive—more engaged) vs email-based (reactive—less engaged) responses suggest the influence of CAD PRS communication may diminish overtime if not reinforced. This engagement effect appears to be more important than the refinement of risk communication, as a stronger association of preventive behavior with degree of CAD PRS is observed in app-based follow-up (Table 5), even though most of this follow-up was completed before the 2019 update and app enhancements. Other barriers to adoption of lipid lowering therapy that we did not measure include: (1) whether study participants believe lipid lowering therapy will reduce their risk, (2) whether study participants believe lipid lowering therapy will induce unwanted side effects, and (3) whether financial or other barriers exist in the access to lipid lowering therapy. These barriers may be more easily overcome through engagement with health care professionals or incentive programs.

Third, the rate of study participant follow-up is lower than desired (18.7%), which can lead to biases in trial results. However, we also note that this follow-up rate is substantially higher than similar digital studies, like the Apple Health study, where the follow-up rate is <1%^29^, and that the observed drop-out rate is equal across study groups and the characteristics of study participants and study respondents remain balanced across study arms at follow-up. Moreover, there is no correlation between the CAD PRS and baseline ASCVD risk factor characteristics (due to the highly multifactorial nature of the CAD PRS and the dominating influence of age on PCE risk calculation), and no correlation between the CAD PRS and clinical risk are induced by differences in participant decisions to respond to the follow-up survey (Table 1). In other words, study response was not related to degree of genetic risk and degree of genetic risk is uncorrelated with PCE risk. Thus, our study benefits from the “random” nature of participant stratification by genetic risk, referred to in econometrics literature as a “natural experiment”. In fact, Table 1 indicates a slight bias towards elevated clinical risk in study respondents from the low CAD PRS group. Indeed, we observe a > 10-year elevation of age in the low CAD PRS vs high CAD PRS lipid lowering therapy initiators. Thus, a high CAD PRS is associated with both increased and earlier initiation of lipid lowering therapy, while elevated PCE risk is more likely to drive lipid lowering initiation in the low CAD PRS group. While loss to follow-up may induce biases in study results, these orthogonal signals support the validity of our findings.

Fourth, our primary outcome is self-reported usage of statins and other lipid-lowering treatments. Approximate PCE scores were reported to the majority of study participants using qualitative risk factor responses. Whether adequate lipid lowering is achieved, or whether self-reported initiation leads to durable adherence is unknown. Our findings should be replicated in additional studies including objective and/or EHR-based outcomes.

On the other hand, several issues also bias us against observing a signal in this study, especially the changing landscape overtime as this study was conducted. Individual level changes in the score^30^, as well as access to different capabilities may have influenced the consistency of our results. Importantly, much of the study follow-up overlaps with the ongoing SARS-CoV-2 pandemic, which has reportedly decreased the use of primary care interventions overall, including cholesterol testing^31^. We expect these issues have diminished our ability to detect the alignment we observe between the CAD PRS and risk reducing actions.

Overall, we find that communication of a high CAD PRS was associated with increased and earlier initiation of lipid-lowering. Further objective, long-term follow-up studies are required to confirm this finding and to dissect the role of CAD PRS communication in adherence to and intensification of lipid-lowering therapy in individuals taking sub-optimal statin dose or other lipid-lowering medications. Longer term objective studies are required to determine whether communication of a CAD PRS can extend its influence from initiation to intensification, adherence, and persistence of lipid-lowering therapy, as well as improved hard outcomes. Finally, we suggest that PRS utility studies should be executed in indication-specific contexts to accurately gauge their influence on early prevention.

## Methods

### Overview

The study protocol “MyGeneRank” (NCT03277365) was approved by the Scripps IRB Protocol #: IRB-16-6835. Informed consent was collected electronically in the study app. Individuals were consented for publication at enrollment. This research conforms with the principles of the Helsinki Declaration.

**Fig. 2:**
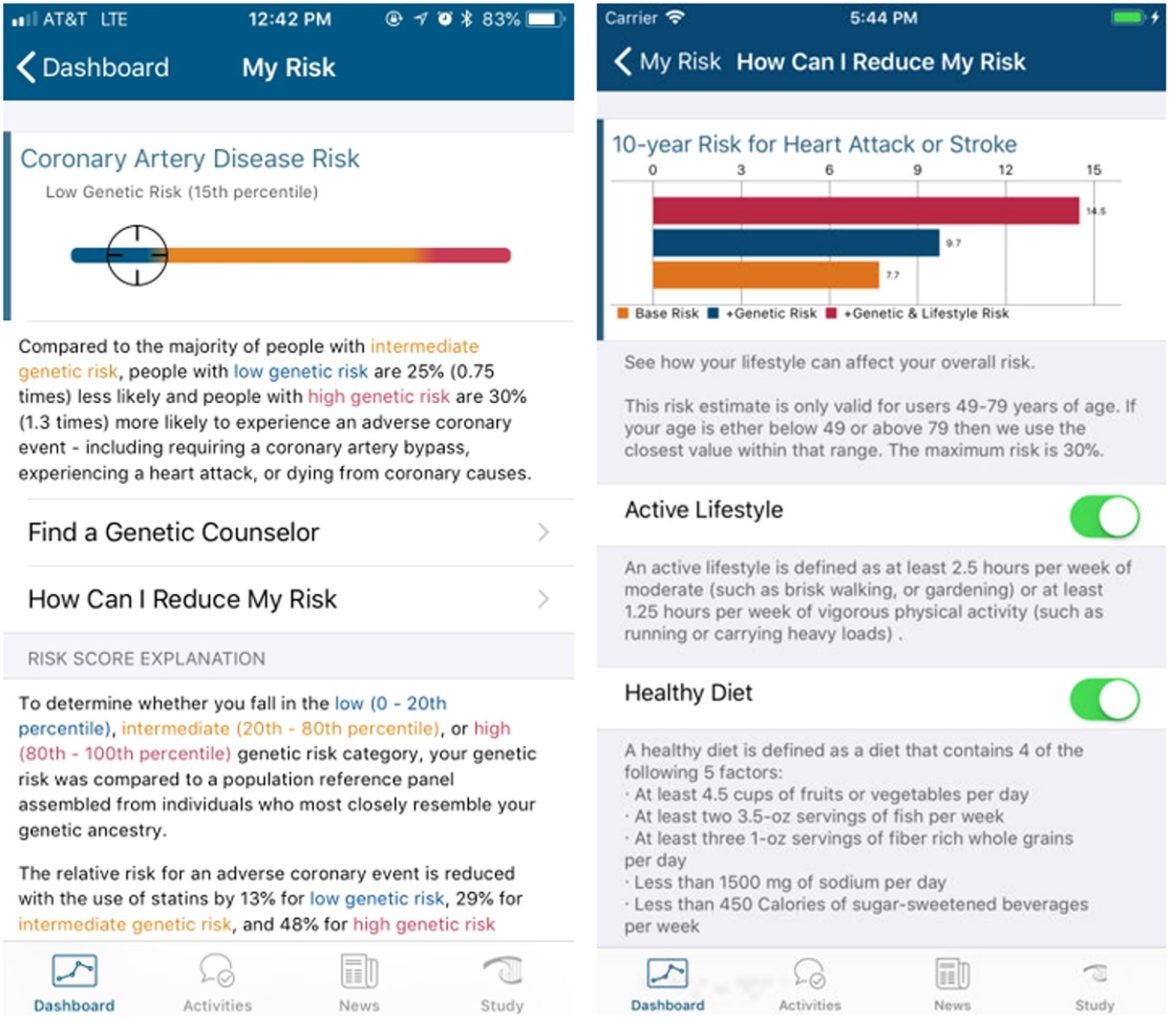
MyGeneRank Screenshots. The two central screens depicted return of CAD PRS results alone (left) as well as the integration of the CAD PRS with 10-year clinical risk in a dynamic risk-reducing interface.

The MyGeneRank study was launched in August of 2017 as solely an iOS application available in the USA. The initial development and basic design is described previously^32^. The first version of the study app calculated and returned a 57-SNP CAD PRS based upon the latest CAD GWAS meta-analysis at the time^33^. An update was launched in December 2019 expanding the application to Android, adding Spanish language, adding a genetic counseling option, and improving the overall interface. The CAD PRS was also updated to calculate and return a 163-SNP CAD PRS based upon the prior score plus the latest efforts in identifying potential causal alleles^34^ and weights from the latest CAD GWAS meta-analysis^35^. The derivation and calculation of these PRSs are described in Supplementary Note 1 and has been previously described in detail^30^. Screenshots of the app interface at launch are presented in Fig. 2 and the current app interface can be viewed at the study website: mygenerank.scripps.edu. All participants were presented with their new score after the update. Most scores did not change substantially after the update, details of which can be found here: mygenerank.scripps.edu/blog/post/mgr-new-update.

### Study tasks

Adults (≥18 years old) with access to a smartphone and existing genetic data from 23andMe were eligible to participate in the MyGeneRank study. Participants download the study application and are presented with an eligibility screen and series of screens summarizing the consent, a complete consent form, HIPAA authorization, and participant bill of rights. PDF copies of these documents are available to study participants within the app, and participants may withdraw from the study at any time. Study participants are asked to link their 23andMe genetic data via API, optionally provide access to their mobile health data via Apple Health and Google Fit, and answer three survey questionnaires. These questionnaires include: (1) a 28-question initial demographic and CAD health and history survey answered prior to the return of results, (2) a 14-question psychosocial survey delivered immediately after the return of results aimed at gauging study participant intentions and feelings in response to their CAD PRS, and (3) a follow-up CAD health survey with questions overlapping with the initial health and history survey. The follow-up survey is made available to study participants in the study app at 6-months post-results but could be answered at any time after 6-months post-results. Follow-up responses were also obtained via email-based electronic survey disseminated via an email-based outreach campaign from November 2020 to January 2021. Three questions were added to the baseline and follow-up survey, and four questions to the psychosocial survey with the December 2019 update.

### Enrollment and responses

**Fig. 3:**
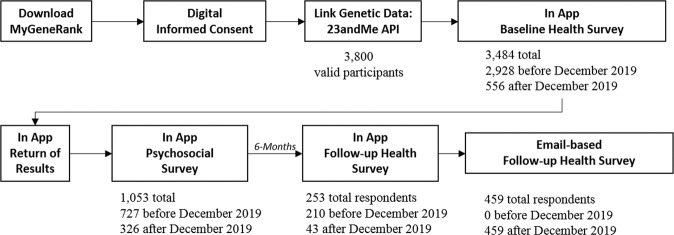
Study flow diagram. Overall study flow and number of participants responding to each survey prior to or before the December 2019 update.

Participants self-referred to the study organically, primarily through word of mouth and social media announcements. A total of 3,800 participants, after duplicate removal, connected their existing 23andMe genetic data with the MyGeneRank system. Approximately, 80% of study participants joined the study prior to the December 2019 update. Of the individuals enrolling prior to the score update, 2.5% responded to the psychosocial survey and 67% responded to the follow-up CAD health survey after receiving their updated score. 253 (6.7%) of participants responded proactively to the app-based follow-up survey. 459 (12%) of participants responded to an email-based follow-up survey after re-contact. Participants were able to skip survey questions, leading to differences in the number of study respondents answering each individual question. Herein, we refer to “study participants” as any enrolled individual. “study respondent” refers to those enrolled individuals whose response includes the final follow-up survey. The study remains open to enrollment with indefinite follow-up, this manuscript is an interim analysis following our time-limited email-based campaign to collect outcomes from current participants. The overall study flow and number of participants responding at each stage is summarized in Fig. 3.

### Risk communication

Study participants view their CAD risk in two interfaces: (1) an interface integrating the CAD PRS with the 10-year ASCVD Pooled Cohort Equations (PCE) derived risk, and (2) a percentile rank view where the percentile rank is calculated relative to simulated individuals of similar genetic ancestry (Fig. 2)^32^. PCE risk is calculated based upon self-reported PCE risk factors. Quantitative measures are used when provided by study participants. Out-of-range values are adjusted to the top or bottom of the range of the PCE calculator as necessary. If a quantitative value is not provided, it is assigned based on survey response to qualitative risk factor questions (as cataloged in Table 1). The assigned values were defined based on guideline-based definitions of these qualitative categories (i.e., high, moderate, and normal/low total cholesterol = 250, 220, and 190 mg/dL, respectively; high, moderate, and normal/low HDL cholesterol = 65, 50, and 35 mg/dL, respectively). If qualitative values are unknown, a normal category value is assigned. Qualitative risk factor responses were >95% complete. Study participants are categorized as low (<20th percentile), average (20th to 80th percentile), or high (>80th percentile) CAD PRS. This quintile-based approach is derived from the foundational Mega et al.^18^ study which established improved statin efficacy for high CAD PRS individuals using these percentile rank thresholds. The number of respondents per question is presented in table headers, where it can be observed that participant and respondent proportions did not significantly deviate from the expected proportions of 20%:60%:20% low:average:high CAD PRS ratios. In other words, survey response was not related to degree of polygenic risk. Any downstream participant actions, including adoption of genetic counseling services, sharing of results with a physician, or any other actions taken outside of score receipt and response to study surveys were unprompted and self-initiated by study participants.

### Comparisons and statistical analysis

For comparisons involving survey responses at baseline vs follow-up, the score the participant received at baseline is used for categorization. For comparisons involving survey responses at a single timepoint, the score (original vs updated) available to the participant at the time of their response was used. For the statin and non-statin lipid-lowering initiation subgroup analysis, the subgroup is defined as those individuals indicating non-use of lipid-lowering therapy in the baseline survey.

Binary choice survey response comparisons are made with a standard one-tailed two-proportion z-test for high vs low CAD PRS individuals. Standard errors for these proportions are a direct function of the provided proportion and sample size. Accordingly, sample sizes per exposure group are provided in each table reporting binary choice survey outcomes. Likert score responses and age comparisons were made using the Mann–Whitney *U* test for high vs low CAD PRS individuals. Likert scores were coded as 1: strongly disagree, 2: somewhat disagree, 3: neutral, 4: somewhat agree, 5: strongly agree. Standard errors are provided in tables providing Likert score outcome comparisons.

Individuals with missing data were discarded on a survey item-by-item basis to maximize sample size for each comparison. Effect modifying variables are internally controlled via “natural randomization” (by CAD PRS status) and the lack of correlation between PCE risk and the CAD PRS, which results in balanced clinical risk factors across comparison groups (see Table 1 for comparisons and “Discussion” for a more detailed explanation of this internal control)^36^.

### Reporting summary

Further information on research design is available in the Nature Research Reporting Summary linked to this article.

## Supplementary information

Supplemental Document

Supplemental Tables S1-S4.

IRB Protocol

Reporting Summary

NPJ DigiMed Checklist

## Data Availability

The raw study data supporting all tables is available as Supplementary Table 1. Participants were consented with assurance that their self-provided individual-level genetic data would not be redistributed. However, individual-level CAD PRS results are available in Supplementary Table 1.

## Code availability

The CAD PRS weight table is available in Supplementary Table 4. This weight table can be used to calculate PRS scores using PLINK or other available PRS calculation packages: https://www.cog-genomics.org/plink/1.9/score.
