# Supplementary material for: Impact of polygenic risk communication: an observational mobile application-based coronary artery disease study
Source: NPJ Digit Med. 2022 Mar 11;5:30. doi: 10.1038/s41746-022-00578-w (PMC8917120; doi:10.1038/s41746-022-00578-w)
Supplement: Supplementary file 1 — Supplemental Document [file 41746_2022_578_MOESM1_ESM.pdf]

Supplementary Note: Polygenic Risk Score (PRS) Derivation for MyGeneRank

Derivation of the Score. An initial 57-SNP score was derived in late 2017 from the Natarajan et al., one of the primary publications establishing enhanced statin benefit in high genetic risk individuals.

Natarajan P, et al. Polygenic Risk Score Identifies Subgroup With Higher Burden of Atherosclerosis and Greater Relative Benefit From Statin Therapy in the Primary Prevention Setting. Circulation. 2017 May 30;135(22):2091-2101.

In 2019 we updated the score using all known CAD risk loci at the time. A review, Erdman et al. 2018, listing 163 loci was used to update the list as the authors had prioritized likely causal alleles. This list is reduced to 161 SNPs when excluding 2 multi-allelic variants.

Erdmann J, Kessler T, Munoz Venegas L, Schunkert H. A decade of genome-wide association studies for coronary artery disease: the challenges ahead. Cardiovasc Res. 2018 Jul 15;114(9):1241-1257.

The intersection of these two scores in terms of loci is shown in Figure 1. The 26 “matched” SNPs are those where the same index SNP is in both lists. 23 proxy SNPs are those where the two scores use different index SNPs but with a minimum R^2 threshold of 0.95. The Erdmann et al. SNP was selected in this case. 8 SNPs are unique to the 57-SNP score. These SNPs were reviewed and confirmed to be associated loci missing from the Erdman et al list. 112 SNPs are unique to the Erdmann et al list. This results in 169 SNPs in the final intersected score. 1 SNP (rs17678683) falls in a narrow recombination region and has no proxy in the Haplotype Reference Consortium – resulting in a final list of 168 SNPs. Weights for all these SNPs are determined from the CardioGram C4D GWAS. Variants in LPA were manually priotized for pathogenicity. The resultant score is returned as a percentile rank normalized to the score distribution expected from an ancestrally matched population derived from the 10000 Genomes Project.

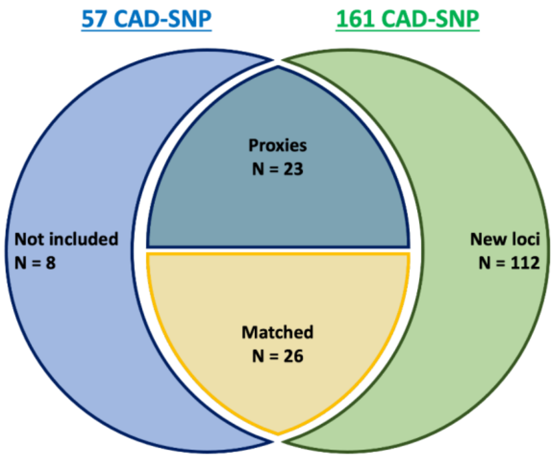

Figure 1. Overlap of 57 and 161-SNP PRSs.

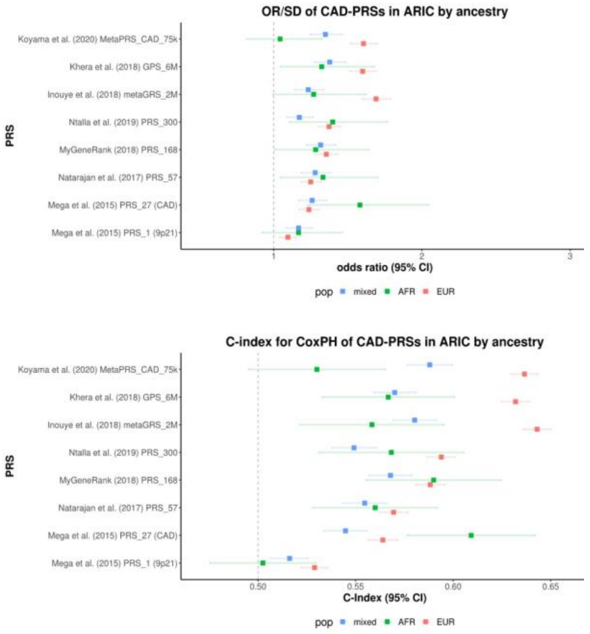

Figure 2. Comparison of CAD PRS predictive performance across ancestries using the ARIC dataset as validation by (top) odds ratio per standard deviation and (bottom) C-index for sex-stratified age-as-time-scale Cox regression.

Generalizable Predictive Performance. We evaluated the generalizability of this score in the Atherosclerosis in Communities Cohort (Figure 2). Individuals are split into European, African, or mixed ancestry based on a 95% cutoff for genetically determined continental ancestry. The predictive performance of our synthetic 168-SNP score is compared against several more simple (Mega et al. and the original Natarajan et al. 57-SNP score) and more complex (Ntalla et al., Khera et al., Inouye et al.) scores. We noted that our score provides equivalent to superior risk stratification in African and admixed individuals, whereas the more complex scores appear to be overfit to the European ancestry populations – which aligns with the eurocentric approach used to generate these scores. In addition, one should note that the more complex scores have not necessarily been validated as associated with improved statin efficacy – thus while predictive performance in European populations is improved, it is uncertain whether improvement in predictive performance actually translates to improved identification of European individuals who would receive greater benefit from statin therapy. Thus, we favor the more simple, generalizable score that is more reflective of initial statin efficacy studies.
