# Supplementary material for: Impact of polygenic risk communication: an observational mobile application-based coronary artery disease study
Source: NPJ Digit Med. 2022 Mar 11;5:30. doi: 10.1038/s41746-022-00578-w (PMC8917120; doi:10.1038/s41746-022-00578-w)
Supplement: Supplementary file 3 — IRB Protocol [file 41746_2022_578_MOESM3_ESM.pdf]

1.0 General Information

1.1 *Please enter the full title of your study:

MyGeneRank

1.2 *Please enter the short title you would like to use as a reference.

MyGeneRank

1.3 Please identify the Research Type?

Genomics

1.4 Please identify the Study Phase?

--none--

2.0 Add Department - Research Site(s)

2.1 List of Department - Research Sites associated with this study:

Primary Dept?	Department Name
•	**Scripps** - Scripps Translational Science Institute

3.0 Assign key project personnel(KSP) access to the project

***The current project status does not allow for changes to the Key Study Personnel. If you wish to change the Key Study Personnel, please contact the IRB.**

3.1 *Please add a Principal Investigator for the project:

Ali Torkamani, Ph.D.

3.2 If applicable, please select the Research Staff personnel:

A) Additional Investigators

[illegible]

- Sub-Investigator
- Nathan E Wineinger, Ph.D.
  Sub-Investigator

B) Research Support Staff

- Emily Spencer, PhD
  Study Coordinator

3.3 *__Please add a Project Contact:__

01. Emily Spencer, PhD
11. Ali Torkamani, Ph.D.

The Project Contact(s) will receive all important system notifications along with the Principal Investigator. (e.g. The project contact(s) are typically either the Study Coordinator or the Principal Investigator themselves).

4.0

Study Interview

Interview Tips:

- ***All questions that require answers are preceded by an asterisk (*). After completing a section click on the 'Save and Continue to Next Section' button in the upper right. If you miss a required question, an error box will appear and the field you missed will be indicated in red.***
- ***You do not have to complete this interview all at one time. If you wish to stop in the middle, any sections you have completed AND SAVED will be saved as a 'Draft' version. You can return to this 'Draft' version by going to the My Studies area of Study Assistant.***
- ***If you are entering a long block of text copied from another source that requires editing, it will be easier to paste this into MS Word on your desktop and do the editing there, then paste into the text box or text editor within this application.***
- ***If you want to go back to a prior section in the interview do NOT use the 'Back' button in the upper right or the 'Back' button in your browser. Click on the section you wish to go back to in the 'Sections' menu on the left. If the 'Sections' menu on the left is not visible, then use the 'Back' button in the upper right. If you do accidentally click on the 'Back' button you will go the Submissions section for your study. Click on the 'Application' link under 'Protocol Items' to return to the interview.***
- ***Help for completing some sections will appear on the right side of the interview. Put your cursor over the bubble containing a question mark and click on the link that pops up to view HELPFUL TIPS.***
- ***When calling or emailing with questions about or problems with this interview please refer to the section title in addition to the section number.***

4.1	***How do you want your Institution, Department, Division, etc. to appear on official IRB Approval Notices?**

Scripps Translational Science Institute

5.0 Independent IRB

5.1	***Are you using a Central/Independent IRB? (*If Yes, be sure to attach the IRB application, sponsor protocol, investigational drug brochure and approved consent/assent forms.*)**

○ Yes ● No

6.0 HDE/HUD

6.1	***Is this a Humanitarian Use Device Registry (HUD)?**

○ Yes ● No

7.0 Exempt/Waived Research

7.1 ***Do you think this research may be Waived under 45 CFR 46.102(f) as '*Not Human Subjects*' research?**
[FOR CLARIFICATION/QUESTIONS, CALL THE IRB OFFICE BEFORE YOU COMPLETE THIS SECTION: 858-652-5500]

Examples that may be Waived include:

- **Use of human derived materials that are purchased from a commerical source**
- **Use of unidentifiable tissue or serum from a biorepository**

(Note: Using or deriving Human Stem Cell lines cannot be waived.)

○ Yes ● No

If Yes, please explain:

7.2 ***(Reminder: If you answered 'Yes' to Waived, please answer 'No' to Exempt.)***

***Do you think your study may be Exempt from IRB review? (*This category is usually only applicable to basic scientists at the Research Institute. If you are not sure, select No. If you are using blood from the Normal Blood Donor service or using or deriving human stem cells, your study is NOT exempt.)*)**

○ Yes ● No

If Yes, please explain in detail:

8.0 TSRI Normal Blood Donor Services

8.1	***Is your ONLY use of human subjects obtaining blood from the TSRI Normal Blood Donor program?**

Note: If you are obtaining any other specimens, answer No.

○ Yes ● No

9.0 Care Line/Co-Management Committee

9.1 *Has your proposal been endorsed by a Scripps Health Care Line or Co-Management Committee?

○ Yes ● No

9.2 If NO, please indicate why not:

This study will not target Scripps Health patients for enrollment and will not recruit or use resources within the Scripps Health medical system.

10.0 Clinical Research Services

10.1 *Is your study being conducted at or through

- **Scripps Clinic**
- **Scripps Green Hospital**
- **Scripps Cancer Center**
- **Scripps Clinical Research Services**

○ Yes ● No

If you answer 'Yes' to this question, your submission will be automatically routed to CRS Director James Mason for sign off before it goes to the IRB.

11.0 Tissue/Blood from Scripps (Patients/Employees) OR Outside of Scripps

11.1 *Are you obtaining blood or tissue from Scripps employees or patients? (*May require informed consent.*)

IF USING THE NORMAL BLOOD DONOR PROGRAM, OR IF THIS IS A CLINICAL TRIAL, ANSWER "NO".

○ Yes ● No

If Yes, please describe:

11.2 *Is your only use of human subjects obtaining blood, tissue, saliva, etc. using collaborators outside of Scripps Health or TSRI? [Check "NO" if your study involves any intervention with human subjects such as drugs, devices, interviews, questionnaires, etc.]

○ Yes ● No

If Yes, please describe:

12.0 Privacy of Health Information and Confidentiality of Data

12.1 ***Will the research involve obtaining individual patient authorization (via patient consent) to use and/or disclose Protected Health Information? *(If No, you must apply for a Waiver of Authorization from the IRB.)***

(Important: If you plan to use Protected Health Information, you must either obtain written authorization from the individual/patient/subject OR request a Waiver of Authorization from the IRB. If you apply for a Waiver, you will also need to provide a description of the data you plan to use. This description can be provided by using the Confidential Data Request form, available on the Scripps intranet.)

● Yes ○ No

13.0 Privacy of Health Information and Confidentiality of Data - Detail

13.1 ***Do you plan to use Scripps Health medical records or patient data to identify potential subjects? *(Note: If Yes, you need to complete a Confidential Data Request form [CDR]. Refer to Scripps policy.)***

○ Yes ● No

13.2 ***What provisions have been made to maintain the confidentiality of the subject's data and/or samples?***

(Important: Identifiable medical information may NOT be stored on non-Scripps electronic devices such as smartphones, laptops, tablets, personal computers, etc. NEVER email any personal identifiers such as name, MR#, etc.)

■ Limited access - IRB must be aware of anyone who has access to identifiable data
□ Stored in secure folder on the Scripps network
■ Research numbers will be assigned. Identification code will be kept separately from the data
■ Password-protected database
□ Other

If Other, please explain:

13.3 ***Will Non-Scripps personnel need to access any Scripps Information Systems to complete the research?***

(Important: Any non-Scripps personnel will require orientation, employee health screening, name badge and IS coordination. They must also go through a vendor/volunteer process before accessing any Scripps data. Policy S-FW-EC-1157 is on the Scripps intranet.)

○ Yes ● No

If Yes, list anyone who will have access to the data that is NOT part of the study staff or sponsoring organization.

**13.4**	***Is there any specific hardware, software and/or transmission of data beyond the standard eCRF?** ***(This would include sponsor- required laptops or software to be loaded onto Scripps PCs, laptops or assets.)***<br><br>**If Yes, please complete the Request for Software Installation or Third Party Application Service Provider (ASP) form.**<br><br>***(Note: Modems are not acceptable.)***

○ Yes ● No

**14.0**	Research Sites and Administrative Review
**14.1**	***Is this a multi-center trial?**

○ Yes ● No

If Yes, are you the Principal Investigator or Program Director for the multi-center trial?

○ Yes ● No

If Yes, (*you are the Principal Investigator or Program Director*), list all non-Scripps sites.

**14.2**	**How will any Non-Scripps sites send data to Scripps Health?**

Participants will send data for study inclusion via Apple ResearchKit.

**14.3**	**What steps have been implemented to verify the integrity of Non-Scripps data prior to loading it into the Scripps network?** ***(Answer is required if Scripps PI is acting as lead site for multi-center study.)***

N/A

**14.4**	***Is the research a project of Scripps Health or the Scripps Research Institute (TSRI)?**

■ Scripps Health - (Conducted by Scripps employees, agents or in Scripps facilities)
■ Scripps Research Institute - (Conducted by TSRI employees, agents or in TSRI facilities)

**14.5**	***Indicate the sites(s) at which data will be collected and/or analyzed. (*Select all that apply.*)**

□ MD Office
■ Outside - Non Scripps Health

- ☐ Scripps Cancer Center (SCC) - Network
- ☐ Scripps Cancer Center - Mercy
- ☐ Scripps Cancer Center - Green
- ☐ Scripps Clinic - Carmel Valley
- ☐ Scripps Clinic - Mission Valley
- ☐ Scripps Clinic - Rancho Bernardo
- ■ Scripps Genomic Medicine (STSI)
- ☐ Scripps Clinic - Torrey Pines
- ☐ Scripps Green Hospital
- ☐ Scripps Memorial Hospital - Encinitas
- ☐ Scripps Memorial Hospital - La Jolla
- ☐ Scripps Mercy Hospital - San Diego
- ☐ Scripps Mercy Hospital - Chula Vista
- ☐ TSRI - Florida
- ☐ TSRI - Normal Blood Donor Service (NBDS)
- ☐ Scripps Radiation/Oncology
- ☐ Scripps Proton Center
- ■ TSRI - The Scripps Research Institute
- ☐ Whittier Institute
- ☐ Scripps Clinical Research Center
- ■ Other

If Other, enter site name.

Virtual, Apple ResearchKit

14.6 Non Scripps and other collaborative research sites.

Please identify additional locations or facilities not listed above.

If using other sites, do they require additional IRB review?

○ Yes ● No

If Yes, what is the status of this other IRB review?

- ○ Not yet submitted
- ○ Pending
- ○ Approved

15.0 Scripps Health Review

15.1 *Does this study involve any Scripps Health facility or Scripps Health patients?

○ Yes ● No

16.0 Clinical Trial

16.1 *Is your project a Clinical Trial?

The NIH defines a clinical trial as a research study in which one or more human subjects are prospectively assigned to one or more interventions (which may

	include placebo or other control) to evaluate the effects of those interventions on health-related biomedical or behavioral outcomes.

● Yes ○ No

**16.2**	**If not a clinical trial, does your project involve testing an assay or device of any sort?**

○ Yes ○ No

**16.3**	***Will your project involve informed consent from individual subjects?**

● Yes ○ No

**16.4**	**If this is a Clinical Trial, what is the NCT number that identifies the trial on www.clinicaltrials.gov?**<br><br>***Note: All clinical trials must be registered in a national database at www.clinicaltrials.gov. Each trial is assigned a unique registry number, the "NCT" number, which begins with NCT followed by an 8-digit number. We must have this number to be able to identify clinical trials ongoing at Scripps, as required by Scripps Health leadership. For commercially-sponsored studies, get the number from the sponsor; for investigator-initiated studies, ask the Principal Investigator or search the clinical trials database to find it.***

□ N/A
■ NCT Not Listed

If NCT number is not listed, explain why:

Pending

17.0 Study Procedures

**17.1**	***Does your study involve any procedures or tests that are NOT considered routine care?**

● Yes ○ No

If Yes, describe all procedures that will be done for Research Purposes ONLY.

Participants will be provided with a Genomic Risk Score and will answer short surveys about what they intend to do with the information and what they actually do with the information.

**17.2**	***Are any of these procedures or tests investigational?**

○ Yes ● No

If Yes, describe how the investigational procedure differs from standard therapy:

18.0 Study Procedures Detail

18.1	*Is the Investigator certified/trained in the use of the procedure(s)?

○ Yes
○ No
● N/A

18.2	*Is the procedure allowed under the scope of practice for staff?

○ Yes
○ No
● N/A

18.3	*Do the principal and other physician investigators have privileges to perform the proposed procedure(s)?

○ Yes
○ No
● N/A

18.4	*Does the proposed research staff have the education and training required to perform the proposed procedures?

□ Yes
□ No
■ N/A

19.0 Drugs

19.1	*Does your study involve the use of any drugs?

○ Yes ● No

20.0 Medical Devices

20.1	*Does your study involve the use of any medical devices?

○ Yes ● No

21.0 Alternative Treatments

21.1	*Are there alternative drug(s), device(s) or procedure(s) that are approved for use in the United States?

○ Yes ● No

If Yes, describe.

21.2	*Is the study drug/device/procedure currently available without participating in

the study?

○ Yes ● No

If Yes, describe:

22.0 Data and Safety Monitoring

22.1 *Describe the plan for monitoring data and safety. ***(A plan is REQUIRED.)***

There is minimal risk to participants as there are no study procedures. Participants will be using genetic data that they already have access to to calculate scores based on publicly available research results.

22.2 *Has a data and safety monitoring committee been set up for this study?

○ Yes ● No

23.0 Blood Draw

23.1 *Will blood be collected specifically for this research?

○ Yes ● No

24.0 Subject Compensation

24.1 *Will subjects receive any payment or compensation for participation in this study?

○ Yes ● No

25.0 Study Plan - Details

25.1 Research Methods - ***Include the Schedule of Events or provide a precise description of the data collection methods. (Attach the Schedule of Events to the Initial Review Submission Form, if applicable)***

Many conditions affecting health are caused by a combination of environment, behaviors, and genes. While we can alter some factors in our lives to reduce our chances of developing different diseases (e.g., not smoking cigarettes), the contribution from our genetic risk encoded by our DNA remains with us throughout our lives. Scientists are still trying to determine the entirety of genetic factors that influence disease, but for some conditions it has been shown that the factors identified thus far can begin to identify people at high to low genetic risk. Looking across the genome, we can calculate a cumulative genetic risk score – which can be used to rank genetic risk compared to other worldwide populations.

The goal of this study is to determine how genetic risk influences health decisions and other things that can be controlled in life. Our first genetic risk score is calculated for coronary heart disease (CAD). CAD ultimately leads to heart attacks, heart failure and sometimes sudden cardiac death and is the main reason heart

disease remains as the number one cause of death worldwide. Other researchers have shown that this genetic risk score can be used to identify people with low, intermediate, and high risk for coronary heart disease. It has also been shown that the use of statins (cholesterol lowering drugs) provides greater benefit and protection against heart attack for people with high genetic risk for coronary artery disease.

Leveraging the Apple ResearchKit and the ResearchKit linked 23andMe API, customers of 23andMe are able to provide researchers access to their genomic data. Participants will use the ResearchKit app to provide consent, view study information, answer surveys, and contact the study team.

Participants will be asked to complete 3 surveys. One before viewing genetic risk scores, one immediately after viewing scores, and one 6 months after viewing scores.

26.0 Study Plan - Clinical Trial

26.1 Clinical Trial Details

***Describe the design of the study (double blind, randomized, etc.) (*Enter N/A if not applicable.*)**

Observational

***Describe any preliminary data that supports or refutes the hypothesis to be tested.**

Mega, et al. Genetic risk, coronary heart disease events, and the clinical benefit of statin therapy: an analysis of primary and secondary prevention trials.Lancet. 2015 Jun 6;385(9984):2264-71.

BACKGROUND:

Genetic variants have been associated with the risk of coronary heart disease. In this study, we tested whether or not a composite of these variants could ascertain the risk of both incident and recurrent coronary heart disease events and identify those individuals who derive greater clinical benefit from statin therapy.
METHODS:

A community-based cohort study (the Malmo Diet and Cancer Study) and four randomised controlled trials of both primary prevention (JUPITER and ASCOT) and secondary prevention (CARE and PROVE IT-TIMI 22) with statin therapy, comprising a total of 48,421 individuals and 3477 events, were included in these analyses. We studied the association of a genetic risk score based on 27 genetic variants with incident or recurrent coronary heart disease, adjusting for traditional clinical risk factors. We then investigated the relative and absolute risk reductions in coronary heart disease events with statin therapy stratified by genetic risk. We combined data from the different studies using a meta-analysis.
FINDINGS:

When individuals were divided into low (quintile 1), intermediate (quintiles 2-4), and high (quintile 5) genetic risk categories, a significant gradient in risk for incident or recurrent coronary heart disease was shown. Compared with the low genetic risk category, the multivariable-adjusted hazard ratio for coronary heart disease for the intermediate genetic risk category was 1·34 (95% CI 1·22-1·47, $p<0{\cdot}0001$) and that for the high genetic risk category was 1·72 (1·55-1·92, $p<0{\cdot}0001$). In terms of the benefit of statin therapy in the four randomised trials, we noted a significant gradient

(p=0·0277) of increasing relative risk reductions across the low (13%), intermediate (29%), and high (48%) genetic risk categories. Similarly, we noted greater absolute risk reductions in those individuals in higher genetic risk categories (p=0·0101), resulting in a roughly threefold decrease in the number needed to treat to prevent one coronary heart disease event in the primary prevention trials. Specifically, in the primary prevention trials, the number needed to treat to prevent one such event in 10 years was 66 in people at low genetic risk, 42 in those at intermediate genetic risk, and 25 in those at high genetic risk in JUPITER, and 57, 47, and 20, respectively, in ASCOT.
INTERPRETATION:

A genetic risk score identified individuals at increased risk for both incident and recurrent coronary heart disease events. People with the highest burden of genetic risk derived the largest relative and absolute clinical benefit from statin therapy.

Describe previous research, pre-clinical or clinical findings that led to the proposed research. (*In early phases of drug or device development where there is little human data, provide the type and number of patients who have received the drug, device or procedure to date.)

As above

***Describe and justify any withdrawal of standard medications or the inclusion of a placebo.**

NA

27.0 Recruitment and Advertising

27.1 *From where will subjects be recruited? Check all that apply:

- ☐ Outpatients
- ☐ Inpatients
- ☐ Your Own Patients
- ☐ Referrals from Other Physicians
- ☐ Hospital or Clinic - Logbooks, schedules, or any other institutional database
- ■ Extramural data or tissue repository or disease database
- ■ Commercial Company
- ☐ Advocacy Groups
- ☐ Private Practice
- ☐ Other

If other, describe.

Participants will be individuals that have used 23andMe personal genomic services AND have an Apple mobile device (iPhone or iPad). Recruitment will not be done through these companies, but potential participants will have to be users of their services/devices.

27.2 *How will subjects be recruited? Check all that apply:

- ☐ Direct contact in a medical setting
- ☐ Direct contact in a non-medical setting (explain)
- ☐ Newspaper Ad (include publication and date)

- ☐ Broadcast media (television/radio/internet)(include details)
- ☐ Posted Notice (location(s))
- ☐ Dear Valued Patient Letter (use the template located in Help - Click on the '?' icon in the upper right and it appears in the list)
- ☐ Newsletters (attach copy or Web site)
- ☐ Flyers
- ☐ Recruitment Organization(s)
- ☐ Dear Colleague Letters
- ■ Social Media
- ☐ Other

Provide additional information for any items checked above (if applicable):

Announcements about the study will be made through social media and scientific resources/journals. Potential participants may also learn about the study through the Apple AppStore.

27.3 *Do you already have a list of potential subjects for this study?

○ Yes ● No

27.4 *Who will do the recruiting? (*Check ALL that apply.)*

- ■ Investigator
- ■ Study Staff
- ☐ Recruiting Agency
- ☐ CRO - Clinical Research Organization
- ☐ Sponsor
- ■ Other

If other, describe.

Study will be announced in social media or participants may encounter it through the Apple AppStore.

27.5 If a patient qualifies for more than one study, how will the Principal Investigator determine which study will be offered to the patient?

N/A

27.6 What limit will be placed on the number of consent forms that a patient will be expected to read and understand at any one time?

N/A

28.0 Alteration of Informed Consent

28.1 *Are you requesting alteration of the informed consent process?

● Yes ○ No

28.2 *Are you requesting permission to waive in-person consent?

● Yes ○ No

If Yes, how will informed consent be obtained?

Non witnessed consent will be obtained through the ResearchKit app as established by Apple protocol. This method is in practice for several studies conducted through ResearchKit.

29.0 Consent Procedure

29.1 *Who will conduct the initial informed consent discussion? *(IMPORTANT: Only personnel who have been added to the protocol in iMedRIS/iRIS and have completed the required education in human subjects protections may obtain informed consent.)*

Informed consent will be obtained through the ResearchKit app. Potential participants will be able to contact study staff to discuss the study if they wish.

29.2 *Describe the experience and qualifications of the person(s) named above.

The ResearchKit app is used for consent for many ongoing studies.

29.3 *Describe the process of obtaining subjects' consent (Include where, when and how the consent will be obtained).

Consent will be obtained via the ResearchKit app after potential participants have downloaded the app. After signing the consent form participants will be able to access the form for printing or viewing at any time from within the app. Potential participants will have the option of contacting research personnel to discuss the study before they sign.

29.4 *Describe the method of documenting that informed consent was obtained.

Once consent has been signed by a participant a pdf of the consent with signature will be saved to a study server.

29.5 *List any and all consent/assent forms that will be used.

MyGeneRankAdultICF

29.6 *Have the consent/assent forms been previously approved by a Non-Scripps IRB?

○ Yes ● No

If Yes, which Non-Scripps IRB? (Including commercial/academic IRBs)

29.7	***How do you plan to inform subjects of new information that might affect their willingness to continue in the study?**

Any updated information or notifications can be posted to the ResearchKit App.

(NOTE: We suggest that new information be incorporated into a simple addendum to the original consent form for enrolled subjects and a revised consent form for new subjects.)

29.8	**If the study involves minors, describe the process of parental permission and how the assent of the minor will be sought.**

Minors will not be enrolled.

29.9	***Will non-English speaking people be approached to participate in this study?**

○ Yes ● No

29.10	**If you need written HIPAA authorizations from subjects, these documents must be retained for at least 6 years.**<br><br>***Note: If research is conducted in a Scripps hospital, a copy of the consent form, including the Authorization, must be filed in the subject's hospital medical record.***

***Check which of the following 3 methods you will use:**

- ■ Retain the entire consent form for 6 years
- □ Retain the Authorization separately for 6 years
- □ Copy the HIPAA Authorization page and send it to Health Information Services
- □ Not Applicable

30.0 Waiver of Privacy Rule Authorization

30.1	***Are you requesting to waive individual Privacy Rule authorization?**

○ Yes ● No

31.0 Risks and Benefits

31.1 *Describe all potential risks of participating in the study, *in simple terms.*

Please include :

- **Risks to the subject's privacy and the confidentiality of data.**
- **The likelihood and seriousness of the most important risks. (Use %, if available, or range, such as 'likely', 'rare', etc.)**
- **If serious risks are involved, explain which risks are expected to be temporary and which might be permanent.**
- **Include the possible consequences of serious risks and possible treatment, if known.**

There is minimal risk to participants as there are no study procedures. Participants will be using genetic data that they already have access to to calculate scores based on publicly available research results.

Only limited PHI will be collected so the risk of loss of privacy is minimal.

31.2 ***Will radiation or radioactive substances be used in your research?** ***For more information on Radiation Safety, move your mouse over the help bubble to the right and click on the link that pops up.***

○ Yes ● No

If Yes, have you submitted the Protocol and Informed Consent form to the Radiation Safety Committee Officer?

○ Yes ○ No

If NO, be advised that you must submit to the Radiation Safety Committee Officer.

31.3 **Describe any use of radiation, including X-rays, fluoroscopy, radioisotopes or protons. Protocols that include any research use of radiation, radioisotopes or protons must be submitted to the Radiation Safety Committee for review.**

N/A

31.4 ***Does the research protocol involve the use of designated HAZARDOUS CHEMICALS in the clinical setting?**

○ Yes ● No

List chemical(s) requiring review.

31.5 **Describe procedures for minimizing any potential risks, including risks to confidentiality, and assess their likely effectiveness.**

PHI will be stored in encrypted databases with restricted access.

31.6 **What provisions have been made for ensuring that medical or professional intervention is available to subjects if an adverse event occurs?**

NA

31.7 ***Is there potential for direct benefit to the subject?**

○ Yes ● No

If yes, describe.

31.8 ***Will there be benefit to the class of subjects or to society?**

○ Yes ● No

If yes, describe.

31.9 *Describe why you think the risks to subjects are reasonable in relation to the anticipated benefits?

There is minimal risk as the participants will not be undergoing any procedures.

32.0 Surveys and Questionnaires

32.1 *Does the project involve the use of Surveys, Questionaires or Interviews?

● Yes ○ No

33.0 Surveys and Questionnaires Detail

33.1 *Will subjects be identified in any way?

● Yes ○ No

34.0 Study Population

34.1 *Briefly describe your targeted population. (*Patients with a condition or disease, healthy control subjects, etc.*)

Study population will be adult individuals that have obtained genetic testing services from 23andMe and own Apple mobile devices. Participants will not be selected based on any other factors.

34.2 *Explain rationale for using human subjects.

This study could not be performed with animals.

34.3 *Age

■ Age Range Not Applicable

Enter the specific age range for study population.

From:

To:

34.4 *Gender

○ Male
○ Female
● Both male and female

34.5 ***How many subjects are you planning to enroll at this institution/site?**

100,000

If this is a chart review, indicate the number of charts: ***(If this is not a chart review, enter 0.)***

0

If necessary, provide explanation below.

This study will be open to as many participants that have used 23andMe and have an Apple mobile device. There are over 1.2 million 23andMe users and hundreds of millions of Apple mobile devices in use.

34.6 ***How many subjects will be enrolled at ALL sites? (Include Scripps and NON-Scripps)**

100,000

If necessary, provide explanation below.

34.7 **To achieve your needed number of subjects, how many subjects do you estimate will need to give informed consent?** ***(Allowing for screen failures)***

unknown

34.8 ***Justification for the number of subjects required:**

While the analysis could be done with a lower number, we will not place restrictions on the number of participants that will enroll as this study will be broadly available in the Apple App Store.

34.9 **Please check all <u>potentially</u> vulnerable populations that are included:**

** Regulated*

- ☐ Children / Minors (subjects less than 18 years) *
- ■ Pregnant Women *
- ☐ Prisoners *
- ■ Economically or educationally disadvantaged persons
- ☐ Non-ENGLISH speaking
- ■ Diminished mental capacity
- ■ Physically disabled
- ■ Students
- ■ Scripps Health Employees
- ■ Scripps Research Institute Employees
- ☐ Other

If other, describe.

If including vulnerable subjects, explain why. Explain what safeguards are included to protect against coercion or undue influence.

Participants will not be selected based on any other criteria than being a user of both 23andMe and an Apple mobile device. Employment status will not be asked or recorded. Individuals must be over 18 to sign up to use 23andMe.

34.10 Inclusion Criteria

***Use the link below to add inclusion criteria.**

Order Number	Criteria
1	Customer of 23andMe willing to share their 23andMe data
1	User of Apple mobile device (iPhone or iPad)

34.11 Exclusion Criteria

***Use the link below to add exclusion criteria.**

Order Number	Criteria
1	Under 18 years old

34.12 Provide justification for inclusion or exclusion of any group (gender, race, ethnicity or other):

No group of people will be excluded.

34.13 Subject Debriefing

Describe any debriefing procedure(s).

None

When will participants be given experimental results and the key to any study blinding?** ***(If not known, request this information from the Sponsor.)

Any study updates will be made accessible to participants via ResearchKit App

35.0 Nursing, Allied Health and Health Services Research

35.1 *Is this Nursing, Allied Health or Health Services Research ?

(Note: Health Services research is the study of the organization, delivery and financing of health care. Some projects of this type may be considered Qualitiy Assurance, Quality Improvement or Process Improvement but NOT research.)

○ Yes ● No

36.0 Human Specimens and Cell Lines

36.1 *Will ANY specimens, other than blood, be obtained for this study?

○ Yes ● No

37.0 Funding Source (*If you are a Principal Investigator receiving a Federal funded grant for collaborative sites to conduct Human Subjects Research, contact the IRB office. You will need to submit IRB documents from the collaborating institution.)*

IMPORTANT: **If ANY funding for this project is coming from a Federal source (federal agency, federal government, National Institutes of Health, National Science Foundation, US military - such as Department of Defense, etc.), the source(s) MUST be entered in this section.**

37.1 *Is this study funded by a commercial sponsor?

○ Yes ● No

37.2 *Is this study funded by a grant?

● Yes ○ No

***Is this an SCMG grant?**

○ Yes ● No

If this study is funded by a grant, are you the PI receiving the grant?

○ Yes ● No

If you are the PI receiving the grant, will any other projects in the grant use human subjects?

● Yes ○ No

If you are the PI for the entire grant, and checked 'Yes' to 'Human Subjects', please submit a copy of the entire grant.

37.3 *Status of funding:

Please select one.

○ Applied/Pending
● Approved
○ Not Applicable

37.4 Sponsor Protocol Number:

37.5 Grant Number:

5 UL1 RR025774)

37.6 ***Granting Agency/Sponsor (You can select more than one agency.)**
(If your agency is not in the list, click on the help bubble to the right.)

If Departmental Funds are being used, click on 'Private' and choose 'Departmental Funds'.

****Note: All studies must have an identifiable source of funding or they cannot be reviewed. Fill in the matrix below.***

	Sponsor	Funding	Protocol Control	Data Coordination	Monitoring	Auditing	Pass Through Funding
**Commercial**							
**Federal or State**	Clinical Translational Science Award	■	□	□	□	□	□
**Private**							
**CRO**							
**Department funds**							

37.7 **Proposed Funding Date - BEGIN**

37.8 **Proposed Funding Date - END**

37.9 **Are part of or all activities in this proposal funded by a training grant?**

○ Yes ● No
