# Supplementary material for: Impact of polygenic risk communication: an observational mobile application-based coronary artery disease study
Source: NPJ Digit Med. 2022 Mar 11;5:30. doi: 10.1038/s41746-022-00578-w (PMC8917120; doi:10.1038/s41746-022-00578-w)
Supplement: Supplementary file 4 — Reporting Summary [file 41746_2022_578_MOESM4_ESM.pdf]

nature portfolio

Corresponding author(s): Ali Torkamani

Last updated by author(s): Dec 9, 2021

Reporting Summary

Nature Portfolio wishes to improve the reproducibility of the work that we publish. This form provides structure for consistency and transparency in reporting. For further information on Nature Portfolio policies, see our Editorial Policies and the Editorial Policy Checklist.

Statistics

For all statistical analyses, confirm that the following items are present in the figure legend, table legend, main text, or Methods section.

n/a	Confirmed	
☐	☒	The exact sample size (n) for each experimental group/condition, given as a discrete number and unit of measurement
☐	☒	A statement on whether measurements were taken from distinct samples or whether the same sample was measured repeatedly
☐	☒	The statistical test(s) used AND whether they are one- or two-sided<br>*Only common tests should be described solely by name; describe more complex techniques in the Methods section.*
☐	☒	A description of all covariates tested
☐	☒	A description of any assumptions or corrections, such as tests of normality and adjustment for multiple comparisons
☐	☒	A full description of the statistical parameters including central tendency (e.g. means) or other basic estimates (e.g. regression coefficient) AND variation (e.g. standard deviation) or associated estimates of uncertainty (e.g. confidence intervals)
☐	☒	For null hypothesis testing, the test statistic (e.g. F, t, r) with confidence intervals, effect sizes, degrees of freedom and P value noted<br>*Give P values as exact values whenever suitable.*
☒	☐	For Bayesian analysis, information on the choice of priors and Markov chain Monte Carlo settings
☒	☐	For hierarchical and complex designs, identification of the appropriate level for tests and full reporting of outcomes
☐	☒	Estimates of effect sizes (e.g. Cohen's d, Pearson's r), indicating how they were calculated

Our web collection on statistics for biologists contains articles on many of the points above.

Software and code

Policy information about availability of computer code

Data collection	A smartphone application platform was used for data collection. We provide data resources required to replicate the primary calculated score (PRS) and all study data collected via electronic survey is provided as supplemental tables and deposited in the Dryad repository with accession provided in the manuscript.
Data analysis	Custom code was not used to perform data analysis.

For manuscripts utilizing custom algorithms or software that are central to the research but not yet described in published literature, software must be made available to editors and reviewers. We strongly encourage code deposition in a community repository (e.g. GitHub). See the Nature Portfolio guidelines for submitting code & software for further information.

Data

Policy information about availability of data

All manuscripts must include a data availability statement. This statement should provide the following information, where applicable:

- Accession codes, unique identifiers, or web links for publicly available datasets
- A description of any restrictions on data availability
- For clinical datasets or third party data, please ensure that the statement adheres to our policy

The raw study data supporting all tables is available as Supplemental Table 1 or at the Dryad repository https://doi.org/10.5061/dryad.t1g1jwt3p Individual-level genetic data is not available for sharing and was not generated with NIH funding.

Field-specific reporting

Please select the one below that is the best fit for your research. If you are not sure, read the appropriate sections before making your selection.

☐ Life sciences ☒ Behavioural & social sciences ☐ Ecological, evolutionary & environmental sciences

For a reference copy of the document with all sections, see nature.com/documents/nr-reporting-summary-flat.pdf

Behavioural & social sciences study design

All studies must disclose on these points even when the disclosure is negative.

Study description	Observational cohort based study with mixed quantitative and qualitative data collection.
Research sample	Any adult self-referring to the study with smartphone access and pre-existing 23andMe genetic data.
Sampling strategy	The study was launched with an indefinite sample size. We accepted all self-referring participants meeting the above requirements.
Data collection	Data is collected by self-report via app-based and email-based surveys.
Timing	Data collection started in August 2017 and stopped in January 2021.
Data exclusions	No participants were excluded.
Non-participation	1232 participants in the high and low CAD risk categories were lost to follow-up.
Randomization	Study participants were randomized by nature, based on their polygenic risk score which is uncorrelated with clinical risk factors.

Reporting for specific materials, systems and methods

We require information from authors about some types of materials, experimental systems and methods used in many studies. Here, indicate whether each material, system or method listed is relevant to your study. If you are not sure if a list item applies to your research, read the appropriate section before selecting a response.

Materials & experimental systems

n/a	Involved in the study	
☒	☐	Antibodies
☒	☐	Eukaryotic cell lines
☒	☐	Palaeontology and archaeology
☒	☐	Animals and other organisms
☐	☒	Human research participants
☒	☐	Clinical data
☒	☐	Dual use research of concern

Methods

n/a	Involved in the study	
☒	☐	ChIP-seq
☒	☐	Flow cytometry
☒	☐	MRI-based neuroimaging

Human research participants

Policy information about studies involving human research participants

Population characteristics	Please see Table 1 for a full description of covariates.
Recruitment	Word of mouth and social media announcements.
Ethics oversight	The Scripps Health IRB approved the study protocol.

Note that full information on the approval of the study protocol must also be provided in the manuscript.
